# Development of a machine learning-based predictive model for long-term adverse outcomes in neonatal bacterial meningitis

**DOI:** 10.1016/j.jped.2025.101472

**Published:** 2025-11-21

**Authors:** Ying Chen, Shengpei Wang, Jing Wu, Chi Wang, Ying Li, Peicen Zou, Ruiqi Xiao, Na Zhang, Huiguang He, Yajuan Wang

**Affiliations:** a Capital Institute of Pediatrics, Department of Neonatology, Beijing, China; b Chinese Academy of Medical Sciences & Peking Union Medical College, Beijing, China; c Laboratory of Brain Atlas and Brain-inspired Intelligence, Institute of Automation, Chinese Academy of Sciences, Beijing, China; d State Key Laboratory of Brain Cognition and Brain-inspired Intelligence Technology, Chinese Academy of Sciences, Institute of Automation, Beijing, China; e Center for Evidence-Based Medicine, Capital Center for Children's Health, Capital Medical University, Capital Institute of Pediatrics, Beijing, China; f Hemangioma and Interventional Vascular Center, Capital Center for Children's Health, Capital Medical University, Capital Institute of Pediatrics, Beijing, China; g Faculty of Pediatrics, Seventh Medical Center of PLA General Hospital, Department of Neonatology, Beijing, China

**Keywords:** Neonates, Bacterial meningitis, Prognosis, Prediction, Machine learning

## Abstract

**Objective:**

To explore the application of machine learning methods for screening risk factors for long-term adverse prognosis in neonatal bacterial meningitis, determine the final prediction model, and evaluate its predictive value.

**Methods:**

This study included 139 full-term neonates diagnosed with neonatal bacterial meningitis in the capital institute of pediatrics between January 2019 and December 2023. Based on follow-up outcomes, they were divided into a poor prognosis group (*n* = 45) and a good prognosis group (*n* = 94). Thirty-three clinical variables were collected. Feature selection was performed using the Least Absolute Shrinkage and Selection Operator, Boruta, and Recursive Feature Elimination. Seven machine learning models were constructed. Model performance was evaluated using metrics including the area under the receiver operating characteristic curve, accuracy, and sensitivity. The Shapley Additive explanation method was used to interpret the models.

**Results:**

Among the seven models, The Random Forest model demonstrates the best overall predictive performance, although Logistic Regression achieved the highest discriminative ability (AUC: 0.903), Random Forest was more suitable for clinical application due to its superior accuracy (0.881), better calibration (Brier score: 0.123), and balanced sensitivity (0.887) and specificity (0.878). Shapley Additive explanation interpretability analysis further revealed that the top three important features were cerebrospinal fluid white blood cell count, cerebrospinal fluid protein levels, and seizures.

**Conclusion:**

Machine learning models, particularly the superior-performing Random Forest, are proven to reliably predict long-term adverse outcomes in NBM patients, aiding in the identification of high-risk individuals. Further validation in broader cohorts is warranted to enhance generalizability and clinical applicability.

## Introduction

Neonatal Bacterial Meningitis (NBM) is an infection of the central nervous system in newborns caused by the invasion of various bacteria. The incidence of NBM varies depending on the region and study population. In developed countries, the overall incidence has decreased to 0.3 cases per 1000 live births, whereas in developing countries it ranges from 0.8 to 6.1 cases per 1000 live births [1]. Globally, an estimated 190,000 newborns die from meningitis annually [2]. Over 50 % of survivors develop long-term neurological sequelae such as deafness, blindness, cerebral palsy, epilepsy, hydrocephalus, or cognitive impairment, among whom one in four experiences severe disability [3].

Poor prognosis in NBM is significantly associated with various high-risk factors, including the host's immune status (e.g., premature birth and low birth weight), severity of intracranial inflammation, pathogen type, and intracranial complications[4, 5, 6]. To reduce confounding from prematurity, the present study specifically enrolled only term infants, focusing on intrinsic prognostic factors of NBM. Traditional prognostic methods rely on single data types and lack multimodal integration, limiting early and precise identification of poor outcomes. Machine learning (ML) can integrate high-dimensional multimodal data and has shown value in neonatal sepsis and necrotizing enterocolitis (NEC). For example, Kausch et al. built a neonatal sepsis model using heart rate and respiration, Area Under the Receiver Operating Characteristic curve(AUROC) > 0.8[7], and Cho et al. identified NEC risk factors in very low birth weight infants with RF and LR (accuracy 0.93; AUROC 0.72)[8].

However, AI studies on NBM remain limited, focusing mainly on diagnosis rather than prognosis. ML offers a promising way to overcome this bottleneck, as shown by Canas et al., combining MRI and clinical data to predict tuberculous meningitis progression (balanced accuracy 0.6) and brain injury (accuracy 0.96)[9]. The team previously used nine ML algorithms to predict short-term outcomes in 433 term neonates with NBM and found that LR achieved a sensitivity 0.541 and specificity 0.974, aiding early intervention. Building on this, the present study aimed to integrate multimodal data and construct an ML model to predict long-term adverse outcomes in NBM, guiding personalized treatment and optimizing resource allocation [10].

## Methods

### Participants

This study included full-term neonates diagnosed with NBM and hospitalized in the Department of Neonatology of the capital institute of pediatrics between January 2019 and December 2023, with follow-up continuing until December 31, 2024. Preterm infants, considered at risk for neurodevelopmental delay, were excluded. This study was approved by the Ethics Committee of the Capital Institute of Pediatrics(Approval Number: SHERLL2024023).

Inclusion criteria: Gestational age ≥ 37 weeks, age ≤ 28 days, and meeting the diagnostic criteria for NBM as follows[11]: 1) Infants with clinical signs of infection, including hypothermia or hyperthermia, poor feeding, apnea, or neurological symptoms such as altered consciousness, seizures, muscle tone abnormalities, irritability, or a bulging fontanel; 2) cerebrospinal fluid (CSF) white blood cell (WBC) count ≥ 20 × 10^6/L, accompanied by elevated CSF protein (> 1.7 g/L) or decreased CSF glucose (< 2.2 mmol/L); 3) positive CSF bacterial cultures or polymerase chain reaction. Meeting the first two criteria allowed a clinical diagnosis, whereas meeting all three criteria confirmed the diagnosis.

Exclusion criteria: 1) Presence of congenital neurodevelopmental malformations, genetic metabolic diseases, or chromosomal abnormalities; 2) history of invasive central nervous system procedures before NBM diagnosis, such as ventriculoperitoneal shunt placement or myelomeningocele repair; 3) intracranial hemorrhage before NBM diagnosis; 4) other types of central nervous system infections; 5) severe asphyxia at birth; 6) patients lost to follow-up

### Follow-up

The follow-up period ended on December 31, 2024, with the shortest follow-up duration being 13 months and the longest being up to 5 years. Patients were followed up through outpatient visits and telephone interviews.

Patients lost to follow-up: ①patients were unreachable immediately after discharge; ②patients failed to complete the minimum one-year follow-up period after one or more initial contacts; ③patients were unable to be reached during a unified long-term follow-up survey conducted in 2024 to assess their most recent outcomes, regardless of their previous follow-up status.

### Grouping

During the follow-up period, the occurrence of any of the following manifestations or death indicated a poor prognosis. Based on the follow-up results, the children were divided into a poor prognosis group (*n* = 45) and a good prognosis group (*n* = 94).

Poor prognosis was defined as the occurrence of any of the following events during the follow-up period: (1) Gesell developmental diagnosis scale screening was conducted for patients attending outpatient follow-up visits, a developmental quotient below 75 in any domain (adaptive, gross motor, fine motor, language, or social behavior); (2) diagnosis of any neurological sequelae (e.g., epilepsy, cerebral palsy, visual, and mental or emotional behavioral abnormalities) by a certified pediatric neurologist according to standard clinical criteria; (3) presence of significant abnormal neuroimaging findings (e.g., hydrocephalus, encephalomalacia, delayed myelination) as reported by a radiologist; or (4) death. All assessors who administered the Gesell Scales were trained and certified to ensure inter-rater reliability. Diagnoses of neurological conditions were made by senior pediatric neurologists.

### Observational clinical variables

The authors collected 33 variables within 7 days of onset: general information (sex, cesarean section, birth weight, age at onset, early-onset NBM); key clinical signs (abnormal temperature, seizures, poor feeding, altered consciousness, vomiting/frothing, cyanosis, bulging fontanelle, widened sutures, hepatomegaly, gazing, tone/reflex abnormalities, jaundice, omphalitis, need for ventilation or inotropes); laboratory tests (C-reactive protein (CRP), peripheral WBC, neutrophil %, hemoglobin, platelet count(PLT), CSF WBC, glucose, protein, culture; blood culture); hearing screening results and cranial MRI findings (ventriculitis, hemorrhage, hydrocephalus, subdural effusion, abscess, encephalomalacia)

### Feature selection

Variables with > 10 % missing were excluded; continuous features were Z-score standardized. The dataset was split into training (70 %) and testing (30 %) with stratified sampling. The authors combined three methods — LASSO (10-fold CV), Boruta (RF 500 iterations, *p* < 0.01), and RFE (RF importance ranking) — retaining only features identified by ≥ 2 methods to enhance reliability and mitigate method bias. All analyses were performed with R v4.3.3.

### Construction of ML models and performance evaluation

The authors developed seven ML algorithms — logistic regression (LR), random forest (RF), support vector machine (SVM), eXtreme Gradient Boosting (XGBoost), k-nearest neighbors (KNN), Light Gradient Boosting Machine (LightGBM), and a neural network — using optimized hyperparameters (grid search + 10-fold CV) with AUC-ROC as the main metric. Performance was validated on the test set by AUROC, precision-recall AUC, accuracy, sensitivity, specificity, PPV, NPV, Brier score, calibration-in-the-large, and decision curve analysis(DCA, threshold 0.05–0.30).

The authors maximized Youden’s index to define model-specific optimal cutoffs and bootstrapped 95 % CIs from 1000 resamples. Python scikit-learn, XGBoost, and LightGBM packages were used.

### Model interpretability

The authors applied Shapley Additive Explanations (SHAP) to quantify feature contributions to predictions, providing global and local interpretability across models using Python SHAP v0.46.0[12].

### Statistical methods

Continuous data were described as mean ± SD or median (IQR); normality and variance were tested with Shapiro–Wilk and Levene’s tests. Student’s *t*-test or Mann–Whitney *U* test compared continuous variables; chi-square test compared categorical variables; *p* < 0.05 considered significant. All statistical analyses were performed with R software (version 4.3.3; R Foundation for Statistical Computing, Vienna, Austria).

## Results

### Characteristics of the study cohort

**Figure 1:**
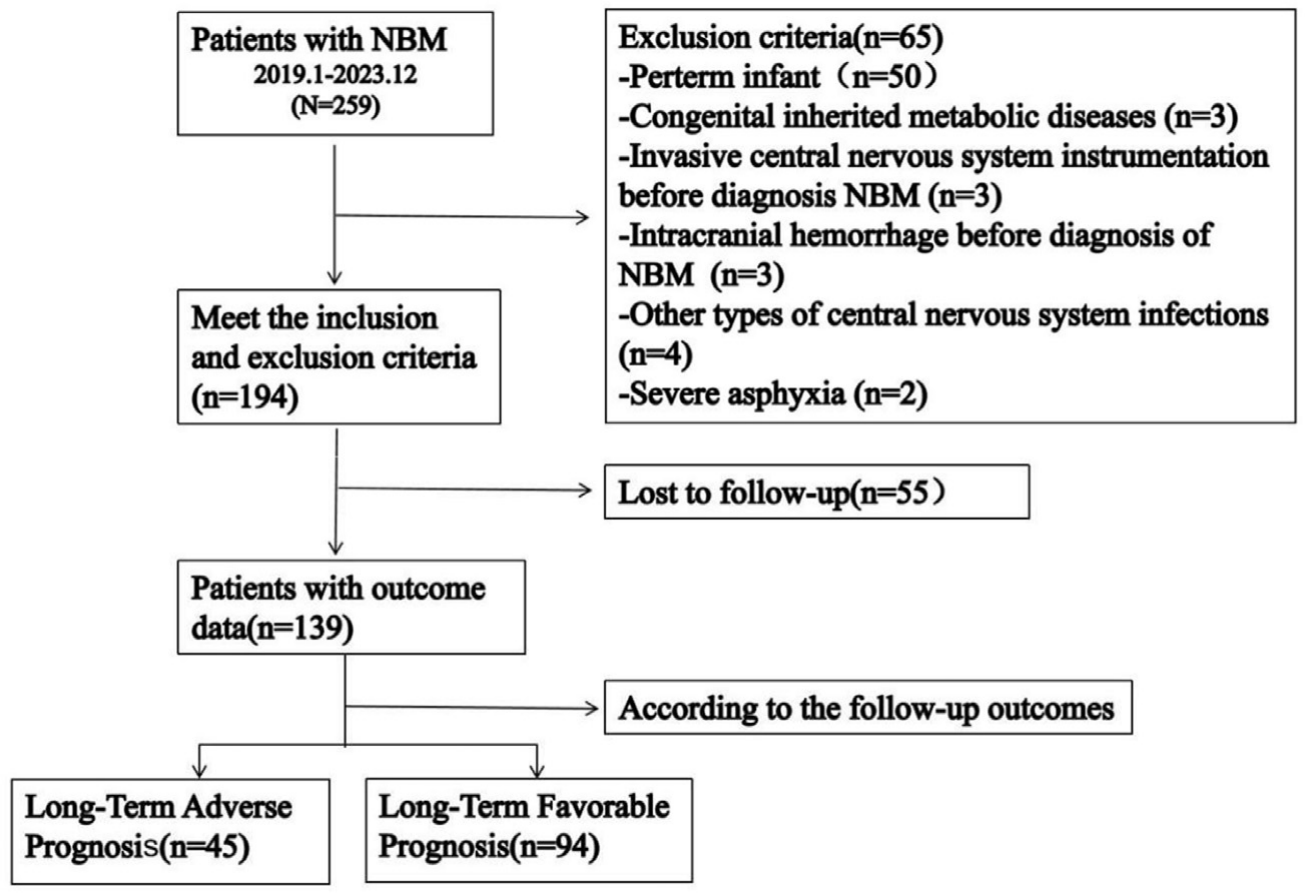
Concise flow chart demonstrating the subject screening steps and grouping.

Between January 2019 and December 2023, 259 infants were diagnosed with NBM; 55 (21.2 %) were lost to follow-up, and 65 were excluded per criteria, and 139 infants were analyzed. Figure 1 shows the study flow chart and patient selection.

NBM, neonatal bacterial meningitis.

Among the 45 infants with poor prognosis, long-term neurological sequelae occurred in 40 (28.8 % of cohort), totaling 73 events: epilepsy (12), developmental delay (15), hydrocephalus (8), language impairment (13), motor dysfunction (16), hearing impairment (3), cognitive/emotional disorders (3), cerebral palsy (1) The distribution of complications per child was: one complication in 19 children, two in 9, three in 10, four in 1, and six in 1

### Baseline characteristics of the NBM group

Significant differences (*p* < 0.05) between groups included neutrophil ratio, CRP, CSF WBC count, protein, culture; seizures; poor feeding; altered consciousness; cyanosis; bulging fontanelle; gazing; muscle tone abnormalities; abnormal primitive reflexes; failed hearing screening; imaging abnormalities; and mechanical ventilation. No significant differences were found in birth weight, hemoglobin, platelet, age at onset, CSF glucose, cesarean section, early onset NBM, sex, blood culture, abnormal body temperature, vomiting/frothing, widened sutures, hepatomegaly, jaundice, omphalitis, or hypotension.as shown in Table 1.

**Table 1:** Baseline characteristics of the study group.

Variables	Total (*n* = 139)	Favorable Prognosis Group (*n* = 94)	Adverse prognosis Group (*n* = 45)	Statistic	p
Birth Weight (kg)	3.48 ± 0.47	3.47 ± 0.50	3.52 ± 0.42	*t*=−0.59	0.559
Sex, n(%)				χ² = 1.52	0.218
Male	92 (66.19)	59 (62.77)	33 (73.33)		
Female	47 (33.81)	35 (37.23)	12 (26.67)		
Cesarean Section (n,%)	66 (47.48)	46 (48.94)	20 (44.44)	χ² = 0.25	0.620
Age at Onset (d)	15.00 (7.50, 20.84)	15.00 (8.00, 19.70)	14.75 (6.00, 22.00)	*Z* = −0.23	0.822
Early Onset NBM (n,%)	8 (5.76)	3 (3.19)	5 (11.11)	χ² = 2.21	0.137
Abnormal body temperature (n,%)	114 (82.01)	80 (85.11)	34 (75.56)	χ² = 1.88	0.170
Seizures (n,%)	34 (24.46)	11 (11.70)	23 (51.11)	χ² = 25.58	<0.001
Poor feeding (n,%)	52 (37.41)	25 (26.60)	27 (60.00)	χ² = 14.50	<0.001
Altered consciousness (n,%)	76 (54.68)	46 (48.94)	30 (66.67)	χ² = 3.86	0.049
Vomiting or Frothing (n,%)	32 (23.02)	20 (21.28)	12 (26.67)	χ² = 0.50	0.480
Cyanosis (n,%)	12 (8.63)	4 (4.26)	8 (17.78)	χ² = 5.44	0.020
Bulging fontanelle (n,%)	17 (12.23)	7 (7.45)	10 (22.22)	χ² = 6.19	0.013
Widened cranial sutures (n,%)	9 (6.47)	5 (5.32)	4 (8.89)	χ² = 0.19	0.666
Hepatomegaly (n,%)	5 (3.60)	1 (1.06)	4 (8.89)	χ² = 3.35	0.067
Gazing (n,%)	13 (9.35)	5 (5.32)	8 (17.78)	χ² = 4.20	0.040
Muscle tone abnormalities (n,%)	21 (15.11)	5 (5.32)	16 (35.56)	χ² = 21.69	<0.001
Abnormal primitive reflexes (n,%)	22 (15.83)	5 (5.32)	17 (37.78)	χ² = 24.07	<0.001
Jaundice (n,%)	63 (45.32)	40 (42.55)	23 (51.11)	χ² = 0.90	0.343
Omphalitis (n,%)	22 (15.83)	18 (19.15)	4 (8.89)	χ² = 2.40	0.121
Failed the hearing screening(n,%)	24 (17.27)	12 (12.77)	12 (26.67)	χ² = 4.12	0.042
Imaging Abnormalities(n,%)	41 (29.50)	14 (14.89)	27 (60.00)	χ² = 29.77	<0.001
Mechanical ventilation (n,%)	9 (6.47)	1 (1.06)	8 (17.78)	χ² = 11.41	<0.001
Hypotension requiring inotropes (n,%)	4 (2.88)	3 (3.19)	1 (2.22)	χ² = 0.00	1.000
WBC (× 10^9^/L)	13.60 (10.07, 18.65)	13.45 (9.55, 18.67)	14.70 (11.20, 18.00)	*Z* = −0.48	0.628
Neutrophil ratio (%)	56.07 ± 15.95	54.13 ± 16.00	60.13 ± 15.21	*T* = −2.10	0.037
Hemoglobin (g/L)	149.29 ± 30.53	148.50 ± 27.88	150.93 ± 35.74	*t* = −0.44	0.662
PLT (× 10^9^/L)	288.24 ± 157.79	300.77 ± 152.36	262.09 ± 167.30	*t* = 1.36	0.177
CRP (mg/L)	10.00 (4.00, 48.50)	5.00 (4.00, 27.50)	27.00 (5.00, 84.00)	*Z* = −3.31	<0.001
CSF WBC Count(× 10^6^/L)	160.00 (44.00, 608.50)	76.50 (40.00, 224.50)	870.00 (184.00, 2500.00)	*Z* = −4.80	<0.001
CSF Glucose (mmol/L)	2.67 (2.15, 2.84)	2.65 (2.21, 2.83)	2.68 (1.80, 3.04)	*Z* = −0.53	0.595
CSF Protein (mg/L)	1464.00 (931.50, 2732.09)	1126.50 (908.50, 1706.96)	3000.00 (1176.00, 4215.00)	*Z* = −4.53	<0.001
CSF Culture(n,%)	12 (8.63)	3 (3.19)	9 (20.00)	χ² = 8.87	0.003
Blood Culture(n,%)	23 (16.55)	13 (13.83)	10 (22.22)	χ² = 1.55	0.213

t, *t*-test, Z, Mann-Whitney test, χ², Chi-square testS, D, standard deviation, M, Median, Q₁, 1st Quartile, Q₃, 3st Quartile. NBM, neonatal bacterial meningitis; WBC, white blood cells, PLT, platelet, CSF, cerebrospinal fluid.

### Feature selection results for NBM

**Figure 2:**
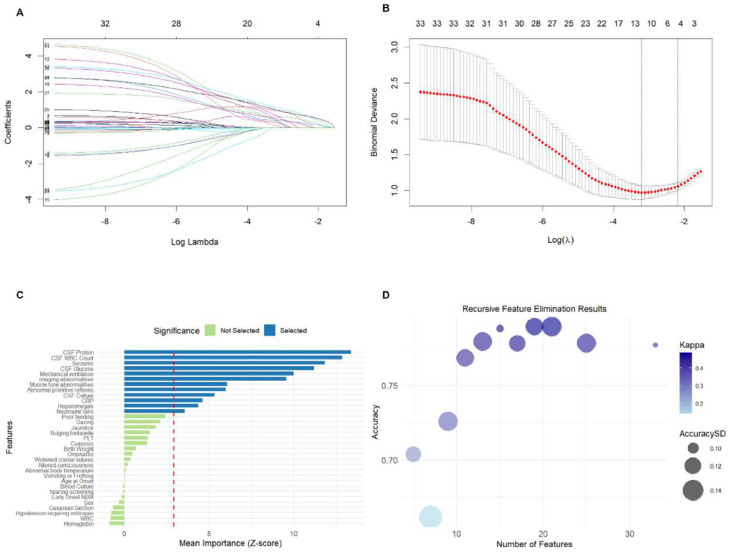
Feature selection with LASSO regression. A: Graph of coefficient paths for LASSO regression; B: Cross-validation plot for LASSO regression; C Feature selection with Boruta. Z-Score ranking of clinical parameters. Variables having box plot in blue are important, and in green as rejected. D Feature selection with RFE Visualization of RFE results can be seen that when 19 features are selected, the model achieves the best performance. LASSO, the Least Absolute Shrinkage and Selection Operator; Recursive Feature Elimination (RFE).

LASSO identified six predictors; Boruta retained 12; RFE retained 19. Five variables (CSF protein, seizures, abnormal primitive reflexes, imaging abnormalities, and mechanical ventilation) were consistent across all methods. Based on ≥ 2-method consensus, 13 variables entered model development. Feature selection results for NBM are shown in the Supplementary Table and in Figure 2A–D.

### Results of the seven model construction and evaluation

**Figure 3:**
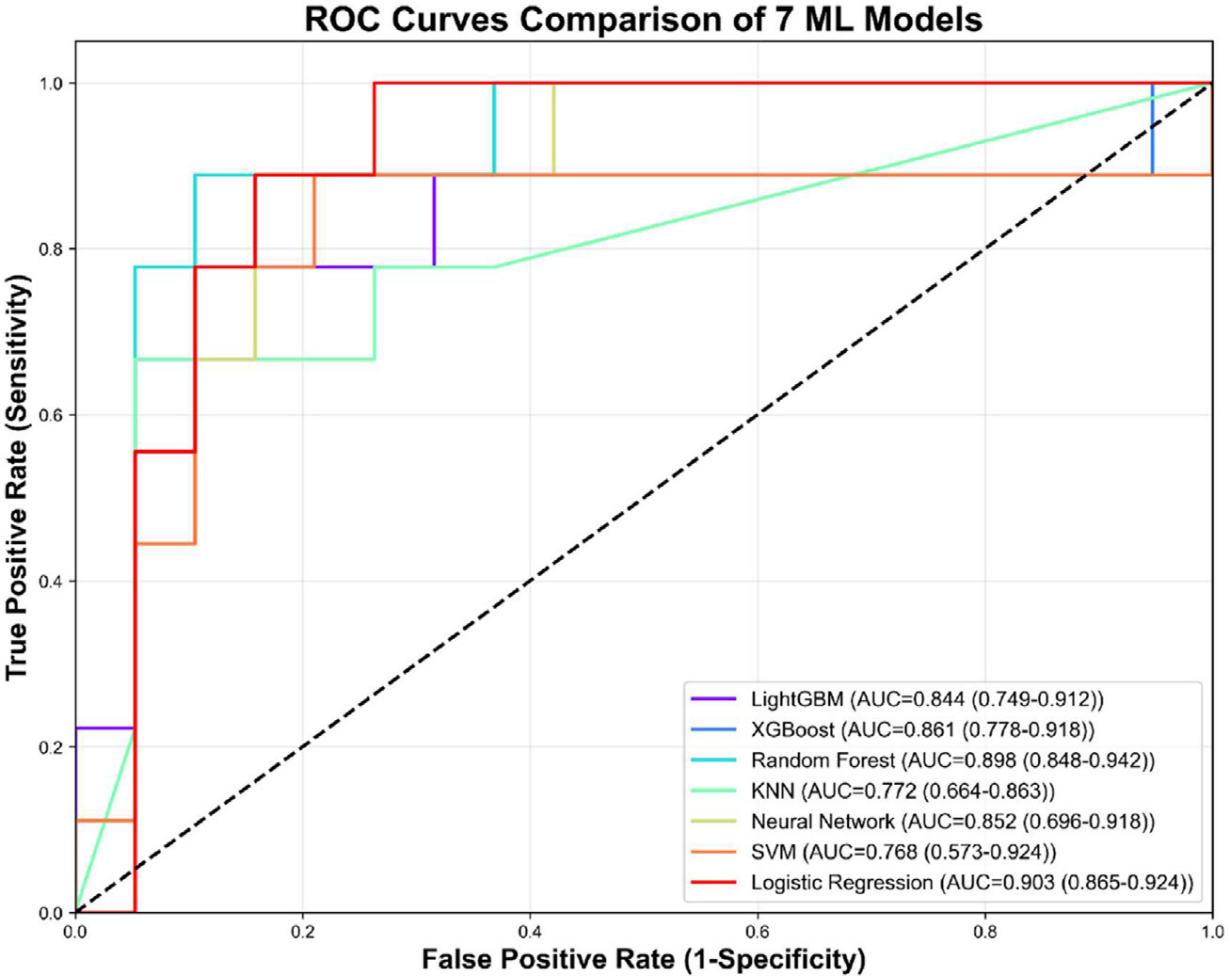
The Receiver operating characteristic curves for predicting Long-term adverse outcomes of NBM of 7 ML models.

AUC, area under curve; LightGBM, Light gradient boosting machine; XGboost,eXtreme gradient boosting machine; RF, Random forest; KNN, k-nearest neighbors; SVM, support vector machine.

The comparative analysis revealed significant performance variations among the seven ML algorithms (Table 2, Figure 3). In terms of discriminative ability, the LR achieved the highest AUROC (0.903, 95 %CI 0.865–0.924) but had poor calibration (Brier 0.235) and lower specificity (0.828). RF showed the best overall profile with AUROC 0.898 (95 %CI 0.848–0.942), accuracy 0.881 (95 %CI 0.821–0.929), balanced sensitivity 0.887 and specificity 0.878, and the lowest Brier score 0.123 (95 %CI 0.103–0.148). XGBoost ranked third (AUROC 0.861) with good calibration (Brier 0.143). LightGBM also performed well (Brier 0.147). KNN and SVM showed unstable decision curves and lower net benefit; the neural network showed intermediate performance.

**Table 2:** Comparison of the performance of the 7 ML models.

Model	AUROC	Accuracy	Sensitivity	Specificity	PPV	NPV	PR AUC	Brier Score	Calibration in Large
LightGBM	0.844	0.825	0.830	0.823	0.712	0.917	0.713	0.147	−0.005
XGBoost	0.861	0.838	0.860	0.827	0.720	0.931	0.725	0.143	−0.01
RF	0.898	0.881	0.887	0.878	0.787	0.945	0.777	0.123	−0.026
KNN	0.772	0.802	0.736	0.833	0.690	0.873	0.641	0.191	−0.09
Neural Network	0.852	0.829	0.895	0.797	0.692	0.948	0.629	0.164	−0.056
SVM	0.768	0.818	0.773	0.840	0.723	0.893	0.662	0.171	−0.004
LR	0.903	0.862	0.936	0.828	0.733	0.969	0.702	0.235	0.174

LightGBM, light gradient boosting machine; XGBoost, eXtreme gradient boosting; RF, Random Forest; KNN, k-nearest neighbors; SVM, support vector machine. LR, logistic regression; AUROC, area under the curve; PPV, positive predictive value; NPV, negative predictive value; PR, precision-recall; AUC, area under the curve.

**Figure 4:**
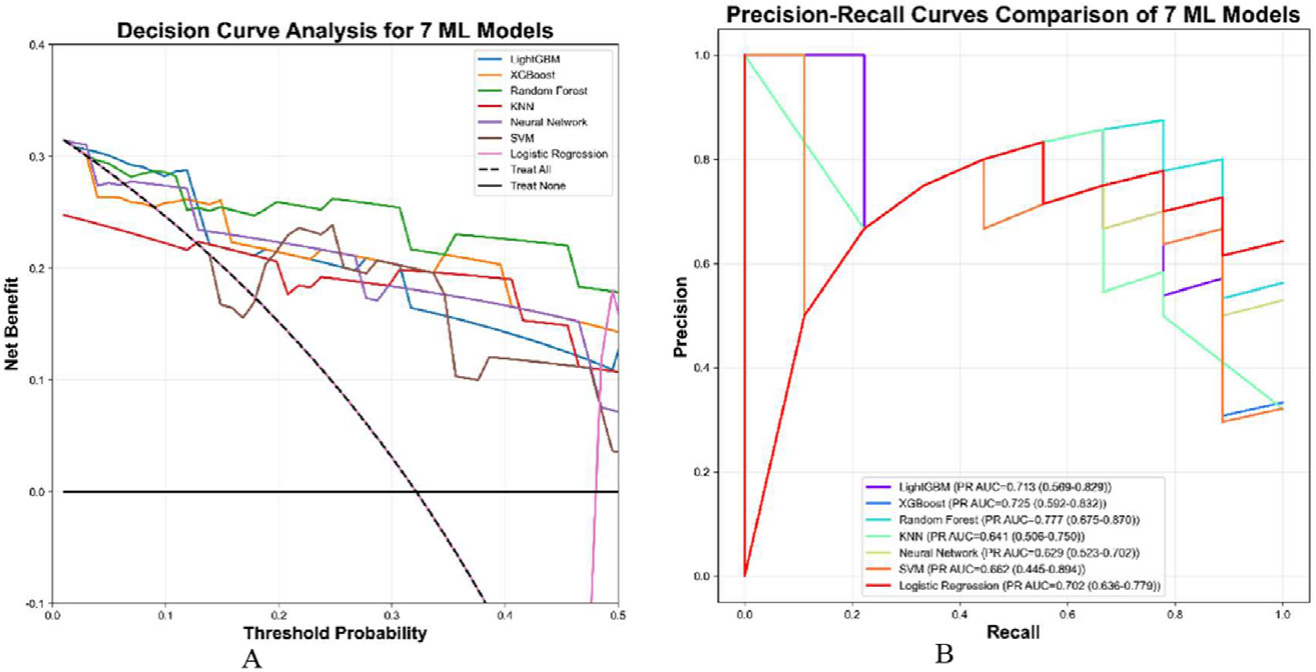
The precision of DCA and PR curves for seven models. (A) Decision Curve Analysis (DCA) for seven models- Precision: Within the reasonable range of threshold probabilities, a higher position of the model curve indicates greater net benefit from clinical decisions using the model at that threshold. (B) Precision-Recall Curve (PR Curve): A curve closer to the upper-right corner signifies better model performance in achieving high precision and recall simultaneously. ML, machine learning.

As shown by DCA (Figure 4A) RF, XGBoost, and LightGBM provided greater net benefit than “treat-all” or “treat-none” within 0.05–0.30 threshold. Precision-recall (PR) analysis (Figure 4B) confirmed RF highest average precision (0.777, 95 %CI 0.675–0.870), followed by XGBoost (0.725); SVM and KNN lowest (0.641–0.662); neural network poorest PR balance despite intermittent spikes.

### Model interpretability

**Figure 5:**
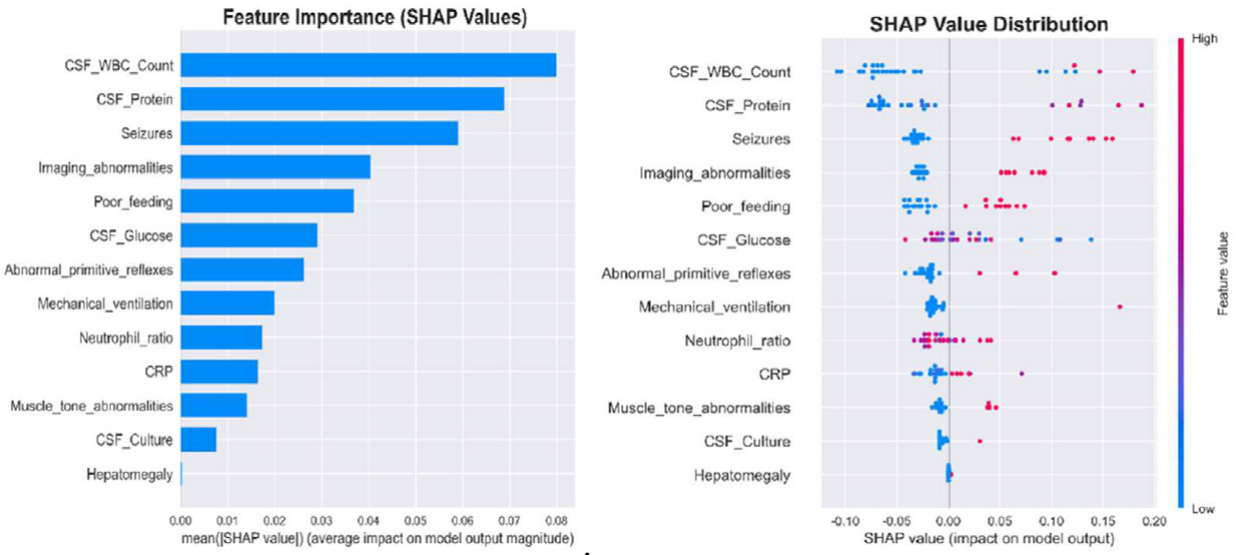
RF Global model explanation by the SHAP method. (A) Feature Importance Scores of the RF Model. The importance of the 13 features was shown in descending order. (B) SHAP summary beeswarm plot of RF Model. The probability of NBM long-term adverse outcomes of NBM increases with the SHAP value of a feature. A dot is made for SHAP value in the model for each single patient, so each patient has one dot on the line for each feature. The colors of the dots demonstrate the actual values of the features for each patient, as red means a higher feature value, and blue means a lower feature value. The dots are stacked vertically to show density. CSF, cerebrospinal fluid CRP, C-reactive protein; WBC, white blood cells; SHAP, Shapley Additive explanations; NBM, neonatal bacterial meningitis; LightGBM, Light Gradient Boosting Machine.

In the RF model, SHAP analysis identified CSF WBC count (mean|SHAP| = 0.080), CSF protein (0.069), and seizures (0.059) as top features. Elevated CSF parameters, seizures, feeding difficulties, and imaging abnormalities increased adverse outcome risk; normal CSF glucose was protective. As shown in Figure 5B.

## Discussion

NBM is one of the most severe central nervous system infections that occurs during the neonatal period. Long-term neurodevelopmental outcomes significantly affect the quality of life of affected children and impose a substantial burden on their families. Compared with traditional univariate analysis methods, this study is, to our knowledge, the first to develop an ML model for long-term prognosis based exclusively on a cohort of term-born infants with NBM.

### ML model performance comparison

This study represents the first comprehensive investigation employing seven distinct ML algorithms to forecast long-term prognostic outcomes in NBM: three conventional models (LR, KNN, and SVM), three ensemble models (RF, XGBoost, and LightGBM), and one deep learning model (a neural network). Based on a multidimensional evaluation framework encompassing discrimination capacity, classification accuracy, calibration, and clinical utility to assess predictive performance, the authors found that the RF model for long-term NBM prognosis prediction demonstrated a combination of strong discriminative ability (AUROC = 0.898), clinically meaningful classification performance with balanced sensitivity (0.887) and specificity (0.878), and exceptional probability calibration (Brier score = 0.123). These findings collectively indicate that the RF model, with its balanced performance across multiple metrics, is more suitable for clinical application than the LR model, despite the latter's superior AUROC. These findings are consistent with those of previous studies on adverse outcome prediction in neonatal disorders, confirming that the RF model is optimal for predicting long-term NBM prognosis. Mentis et al. explored the differential diagnosis of bacterial and viral meningitis using LR, RF, and naive-Bayes algorithms, and found that LR and RF showed the best performance, with over 95 % accuracy for viral meningitis and 78 % for bacterial meningitis[13]. Pinheiro et al. used the LR, KNN, and RF algorithms to diagnose bacterial meningitis and found that the RF model showed the best performance, with a 90.6 % accuracy rate[14]. The authors also found that LR demonstrated superior AUROC (0.903) and sensitivity (0.936), but it was the poorest-calibrated model (Brier score = 0.235). Duci et al. developed a predictive tool for tuberculous meningitis diagnosis based on LR, RF, and regression tree models[15]. Based on these findings, the present study demonstrates that the RF and LR algorithms have emerged as robust prognostic tools for long-term outcome prediction in NBM.

### Key factors for NBM

This study employed three feature-selection methods: LASSO, Boruta, and RFE. Finally, only features identified by at least two methods were retained for the final modeling, enhancing reliability while mitigating method-specific biases. Thirteen key predictors were identified; these predictors are highly consistent with the clinicopathological mechanisms. Among these, CSF WBC count was discarded by LASSO. This was primarily due to the characteristics of the LASSO method (handling collinearity and linear assumptions), which may have underestimated or masked its effects. However, this was consistently identified as a critically important feature by both the Boruta and RFE methods, which are better at capturing complex relationships. This finding demonstrates its central role in predicting adverse outcomes related to NBM. Furthermore, the feature selection approach may have inadvertently favored RF. Although using the intersection of features from LASSO, Boruta, and RFE enhanced stability, the RFE-selected feature set encompassed all features identified by the other two methods. This overlap potentially created an inherent advantage for RF during model training.

SHAP interpretability analysis further revealed that the top three important features are ranked as follows: CSF WBC count and protein levels, and seizures. Among these, the CSF WBC count is the most direct indicator of intracranial infection severity. In this study, the CSF WBC count had the highest weight in the RF model, predicting poor long-term prognosis in NBM. Huang et al. also confirmed its superior diagnostic value compared with parameters like CSF protein and glucose levels, and persistently elevated CSF WBC was significantly associated with poor prognosis. In this study, elevated CSF protein levels ranked second in the RF model's feature importance ranking, demonstrating good discriminatory power for long-term poor prognosis in NBM, which is largely consistent with previous findings[16]. Multiple studies have shown that elevated CSF protein levels are independently associated with poor prognoses in patients with NBM. However, its sensitivity, specificity, and diagnostic thresholds vary significantly, with a wide critical range (1.0–5.0 g/L), potentially influenced by gestational age and postnatal age. Patients with levels > 5.0 g/L have a significantly increased risk of hydrocephalus[17, 18, 19]. Among the clinical manifestations, seizures ranked third in the RF feature importance ranking in this study. Ouchenir et al[19]. found that infants with seizures had a 12-fold increased risk of death at discharge and were more prone to hearing loss, motor impairments

### Innovation and limitations of the study model

Utilizing comprehensive long-term follow-up data, this study is the first to construct an ML prediction model applicable to the long-term prognosis of patients with NBM. By incorporating physiological characteristics specific to the neonatal population — such as early-onset meningitis, full anterior fontanelle, and incomplete elicitation of physiological reflexes — the model requires only 13 routine clinical indicators for risk assessment. By innovatively integrating multiple feature selection methods, the model effectively mitigated the issues of overfitting and variable collinearity inherent in traditional regression analyses. Compared to prediction models relying on complex laboratory tests, this approach is more suitable for the practical conditions of neonatal wards in most hospitals. Furthermore, the study systematically compared seven types of mainstream models and employed SHAP to interpret the optimal model. Through visualization of individualized predictions, it enhanced model transparency and clinical trustworthiness, facilitating the identification of high-risk cases and providing an efficient tool for precise risk stratification in resource-limited settings.

This study has several limitations. First, the relatively small sample size and somewhat high loss-to-follow-up rate may have constrained the performance of the machine learning models. Furthermore, the single-center design limits the external generalizability of the present findings. Future studies should prioritize establishing multicenter prospective cohorts to enhance validation and broader applicability. Second, class imbalance was present, with adverse outcomes accounting for only 32.4 % of the total sample. This may lead to overfitting to the majority class and reduce the model’s ability to identify minority-class cases. Although stratified sampling and threshold optimization using Youden’s index were applied to mitigate this issue, future research could explore advanced techniques such as synthetic minority over-sampling technique Synthetic Minority Over-sampling Technique or weighted loss functions to better handle imbalanced data and improve model robustness. Finally, although the RF model showed the best overall predictive performance with the lowest Brier score (0.123), its calibration-in-the-large value (–0.026) indicated a slight systematic underestimation of risk. This highlights that even models with strong discrimination can exhibit poor calibration. Uncritical reliance on such miscalibrated predictions in clinical settings may lead to underestimation of patient risk and delayed intervention. Future work should emphasize calibration as a core model evaluation criterion alongside discrimination and consider applying post-hoc calibration techniques to improve probabilistic accuracy.

## Conclusion

Based on ML technology, this study developed the first predictive model for the long-term adverse outcomes of NBM. Thirteen core predictive factors were identified by integrating multimodal clinical data and employing a combined feature-selection strategy using LASSO, Boruta, and RFE. Among the seven ML models, the Random Forest model demonstrates the best overall predictive performance. The SHAP interpretability analysis further validated the contributions of the key features. This model provides a reliable tool for the early identification of high-risk infants and guiding individualized interventions, although future validation through multicenter, large-sample cohorts and bedside clinical implementation is required.

## Funding

All data generated or analyzed in this study are included in the published article. This study was supported by the Beijing Natural Science Foundation (nos. 7244289 and 7232009), Beijing Municipal Administration of Hospitals Incubation Program (No. PX2024047), National Natural Science Foundation of China (no. 62201569), and High-level Public Health Technical Personnel Construction Project of the Beijing Municipal Health Commission (Grant no Academic leader: -03-02).

## Ethics statement

The study protocol was approved by the Ethics Committee of Children's Hospital, Capital Institute of Pediatrics (Approval Number: SHERLL2024023)

## Consent

The study involves anonymous data analysis with no risk to participants, and waiver of informed consent is approved by the ethics committee due to minimal impact and impossibility of individual identification.

## Authors’ contributions

Ying Chen: writing–original draft preparation (lead), funding acquisition(lead), project administration (equal), formal analysis (equal). Yajuan Wang: conceptualization (lead), supervision (lead). Shenpei Wang: methodology (lead), funding acquisition(equal), writing–review & editing (equal). Jing Wu: methodology (equal), funding acquisition(equal), writing–review & editing (equal). Chi Wang: investigation(euqal), methodology (equal). writing–original draft preparation (supporting).Ying Li and Peiceng Zou: writing–original draft preparation (equal). Ruiqi Xiao: investigation(euqal), writing–review & editing (supporting),project administration(equal). Huiguang He: Conceptualization (lead), project administration(equal), resources(equal).Na Zhang: writing–review & editing (equal), resources(equal), data curation (equal). data curation (equal),project administration(equal).

## Data availability

The datasets generated and/or analyzed during the current study are not publicly available to protect patient privacy and comply with ethical regulations. However, they can be made available from the corresponding author upon reasonable request, pending approval from the Ethics Committee of the Capital Institute of Pediatrics. In addition, the code used in this study is also available from the corresponding author upon reasonable request.

## Conflicts of interest

The authors declare that they have no known competing financial interests or personal relationships that could have appeared to influence the work reported in this paper.

## Appendix Supplementary materials

Image, application 1
