# Supplementary material for: Development of a machine learning-based predictive model for long-term adverse outcomes in neonatal bacterial meningitis
Source: J Pediatr (Rio J). 2025 Nov 21;102(1):101472. doi: 10.1016/j.jped.2025.101472 (PMC12681912; doi:10.1016/j.jped.2025.101472)
Supplement: Supplementary file 1 [file mmc1.docx]

**JPED-D-25-00283_Supplementary Material**

**Appendix Table 1** Features Selected by LASSO, Boruta and RFE Methods.

|  | Variable Names | LASSO | Boruta algorithm | RFE Analysis | Vote Score |
| --- | --- | --- | --- | --- | --- |
| 1 | Age |  |  |  | 0 |
| 2 | Birth Weight |  |  | Yes | 1 |
| 3 | Cesarean Section |  |  |  | 0 |
| 4 | Early Onset NBM |  |  |  | 0 |
| 5 | Sex |  |  |  | 0 |
| 6 | CRP Level |  | Yes | Yes | 2 |
| 7 | WBC |  |  |  | 0 |
| 8 | Neutrophil Ratio |  | Yes | Yes | 2 |
| 9 | Hemoglobin |  |  |  | 0 |
| 10 | PLT Count |  |  | Yes | 1 |
| 11 | CSF WBC Count |  | Yes | Yes | 2 |
| 12 | CSF Glucose |  | Yes | Yes | 2 |
| 13 | CSF Protein | Yes | Yes | Yes | 3 |
| 14 | CSF Culture |  | Yes | Yes | 2 |
| 15 | Blood Culture |  |  | Yes | 1 |
| 16 | Abnormal Body Temperature |  |  |  | 0 |
| 17 | Seizures | Yes | Yes | Yes | 3 |
| 18 | Poor Feeding | Yes |  | Yes | 2 |
| 19 | Altered Consciousness |  |  |  | 0 |
| 20 | Vomiting or Frothing |  |  |  | 0 |
| 21 | Cyanosis |  |  |  | 0 |
| 22 | Bulging Fontanelle |  |  |  | 0 |
| 23 | Widened Cranial Sutures |  |  |  | 0 |
| 24 | Hepatomegaly |  | Yes | Yes | 2 |
| 25 | Gazing |  |  | Yes | 1 |
| 26 | Muscle Tone Abnormalities |  | Yes | Yes | 2 |
| 27 | Abnormal Primitive Reflexes | Yes | Yes | Yes | 3 |
| 28 | Jaundice |  |  | Yes | 1 |
| 29 | Omphalitis |  |  | Yes | 1 |
| 30 | Failed the Hearing Screening |  |  |  | 0 |
| 31 | Imaging Abnormalities | Yes | Yes | Yes | 3 |
| 32 | Mechanical Ventilation | Yes | Yes | Yes | 3 |
| 33 | Hypotension Requiring Inotropes |  |  |  | 0 |
